# Is the Advent of New Surgical Procedures Changing the Baseline Features of Patients Undergoing First-Time Glaucoma Surgery?

**DOI:** 10.3390/jcm13113342

**Published:** 2024-06-06

**Authors:** Alessandro Palma, Giuseppe Covello, Chiara Posarelli, Maria Novella Maglionico, Luca Agnifili, Michele Figus

**Affiliations:** 1 Department of Surgical, Medical, Molecular Pathology and Critical Care Medicine, University of Pisa, 56126 Pisa, Italy; alessandro93.palma@gmail.com (A.P.); giucovello@gmail.com (G.C.); chiara.posarelli@med.unipi.it (C.P.); m.novella.maglionico@gmail.com (M.N.M.); 2 Ophthalmology, Department of Medical and Surgical Specialties, Azienda Ospedaliero Universitaria Pisana, 56124 Pisa, Italy; 3 Ophthalmology Clinic, Department of Medicine and Aging Science, University G. d’Annunzio of Chieti-Pescara, 66100 Chieti, Italy; l.agnifili@unich.it

**Keywords:** glaucoma, surgical treatment, intraocular pressure, minimally invasive glaucoma surgery, visual field

## Abstract

**Background**: The aim of this study was to determine if the rise in new surgical procedures for glaucoma is changing the baseline features of patients. **Methods**: In this retrospective study, we reviewed the baseline features of patients undergoing their first glaucoma surgery in 2011 and 2021, collecting data regarding intraocular pressure (IOP), visual field (VF) parameters, stage of disease, and the type of surgery. **Results**: In the study, 455 patients were included in the analysis. From these, 230 eyes had glaucoma surgery performed in 2011 (Group A) and 225 eyes in 2021 (Group B). When considering the baseline features, Group A was older than Group B (72.7 ± 10.7 and 70 ± 12.4 years; *p* = 0.02, respectively), and showed a significantly more advanced VF mean defect (−16.4 ± 8.8 and −13.8 ± 8.7 dB; *p* < 0.01, respectively) and a higher IOP (25.9 ± 6.6 and 24.9 ± 7.8 mmHg; *p* = 0.02, respectively). Overall, severe VF damage at the time of surgery was more frequent in Group A (74.3%) than in Group B (60.8%) (*p* < 0.01). The overall number of traditional glaucoma surgeries was 211 in 2011, reducing to 94 ten years later, with similar severe pre-operative VF defects. In 2021, minimally invasive bleb surgery (MIBS) represented 58% of all surgeries. **Conclusions**: In the last ten years, patients receiving glaucoma surgery for the first time were younger, had less severe disease, and a more contained IOP. The baseline feature modifications were probably related to the diffusion of new procedures, especially MIBS, which allowed for treating patients at an earlier stage, reserving traditional procedures for advanced cases.

## 1. Introduction

Glaucoma is a leading cause of irreversible blindness and the second most common cause of blindness worldwide [1,2]. Due to the progressive increase and aging of the population, the number of glaucomatous patients is expected to grow, and it was estimated that by 2040, approximately 111.8 million people will be affected by glaucoma [3]. Glaucoma significantly impacts individuals’ quality of life, posing a substantial public health challenge. The term ‘glaucoma’ refers to a group of different progressive optic neuropathies in which the retinal ganglion cell loss represents the main anatomical hallmark, with corresponding permanent visual field damage [4]. To date, lowering the intraocular pressure (IOP) is the only proven treatment for managing the disease. As known, topical medications and laser treatment allow for controlling the disease in a large portion of patients; however, a significant part of them may require surgery during the course of the disease [5,6].

According to the literature, in the early stages of glaucoma, medical or laser treatment is recommended, while severe or uncontrolled glaucoma requires surgery [7]. Trabeculectomy remains the gold standard surgery, but serious and sight-threatening complications may occur [8,9,10]. In the recent years, with the aim to reduce the post-operative complications of traditional filtration surgery, new devices and procedures have been introduced in clinical practice, leading clinicians into an era of minimally invasive glaucoma surgery (MIGS) [6].

The term MIGS was coined and introduced in 2009 to describe new surgical approaches to lower the IOP, that share the following characteristics: an ab-interno approach, minimal manipulation of the target tissue, efficacy in lowering the IOP, safety from serious complications, a short learning curve for the surgeon, and short recovery time for patients [11]. MIGS procedures work through different ways to reduce the IOP: improving trabecular meshwork outflow or moving the aqueous humor under the conjunctiva, Tenon’s capsule or suprachoroidal space [12]. The rationale behind MIGS lies in its ability to provide moderate IOP lowering with a safer side effect profile, making it particularly suitable for patients with mild-to-moderate glaucoma. Despite their advantages, the effectiveness of MIGS procedures in significantly lowering IOP has been a point of debate, leading to their selective use based on the stage of glaucoma and individual patient needs [7]. In Italy, the available MIGS procedures include: iStent^®^ and iStent^®^ Inject, Hydrus^®^ Microstent, Kahook Dual Blade^®^, GATT (gonioscopy-assisted transluminal trabeculotomy), XEN^®^ Gel, Stent and PRESERFLO^TM^ MicroShunt.

Recently, the term minimally invasive bleb surgery (MIBS) was introduced to represent a well-defined and autonomous subfield of MIGS, which shares the creation of a conjunctival filtration bleb with traditional surgery and the safer surgical profile with MIGS [13,14,15]. Currently, only two MIBS devices are available globally, the XEN^®^ Gel Stent and the PRESERFLO^TM^ MicroShunt. These two procedures, in contrast to other MIGS procedures, are indicated in the moderate and severe stages of glaucoma [16].

Given their higher safety profile, MIGS could induce clinicians to anticipate the timing of surgery, thus potentially offering availability to patients with different clinical characteristics compared to those receiving standard surgery.

To date, whether the rise in MIGS and MIBS is changing the characteristics of patients at baseline, i.e., when the clinicians recommend them surgery to control the disease, has not been specifically investigated in our country. The aim of this study was to evaluate and compare the baseline features of glaucomatous patients at an academic second-level glaucoma center, who underwent their first surgery in 2011, with those receiving their first surgery ten years later, in 2021, to determine if the rise in minimally invasive approaches is changing the baseline features of patients and, thus, our approach to glaucoma surgery timing. In particular, the study aims to evaluate the possible anticipation of surgery in glaucoma patients with the advent of new and safer surgical devices.

## 2. Materials and Methods

### 2.1. Study Design and Patient Population

In this monocentric, retrospective study, data from patients who underwent glaucoma surgery between January and December 2011, and between January and December 2021 were collected. The study was conducted in accordance with the guidelines of the Declaration of Helsinki, and was approved by the Area Vasta Nord Ovest Ethical Committee (CEAVNO) with the code 24294_FIGUS.

All surgeries were performed by two glaucoma surgeons at the Ophthalmology Unit of the Department of Surgical, Medical and Molecular Pathology and Critical Care Medicine, University of Pisa.

The inclusion criteria were as follows: age > 18 years, uncontrolled glaucoma with an IOP > 21 mmHg and progressive glaucomatous damage at the visual field (VF) confirmed on three consecutive examinations [Humphrey field analyzer (HFA) II 750; Carl Zeiss Meditec Inc., Dublin, CA, USA (30-2 test, full threshold)], despite maximal medical topical therapy or intolerance to therapy. Eyes scheduled for combined cataract and glaucoma surgery were also considered eligible.

The exclusion criteria were as follows: previous glaucoma surgery, traumatic glaucoma, and the absence of VF in the two months preceding surgery.

Data collected before surgery included best-corrected visual acuity, Goldmann applanation tonometry, slit lamp biomicroscopy of the anterior segment of the eye, gonioscopy with a 3-mirror contact lens, fundus examination, central corneal thickness, and a 30-2 full-threshold visual field test (HFA). The pre-surgical IOP value had to be obtained as the mean of the three measurements taken at 9:00 a.m. of day 7, 1 and at the day of surgery. Glaucoma staging was defined using the Hodapp–Parrish–Anderson classification (HPAc) as early, moderate, and severe [17]. The VF parameters were obtained from the last perimetry performed no more than two months before surgery. The included glaucoma subtypes were as follows: primary open-angle glaucoma (POAG), primary angle-closure glaucoma (PACG), pseudoexfoliation glaucoma (PXG), pigmentary glaucoma (PG), normal-tension glaucoma (NTG), neovascular glaucoma (NVG), ocular hypertension (OHT), and uveitic glaucoma.

The following glaucoma surgeries were included in the study: trabeculectomy with mytomicin-C (trabeculectomy), Ahmed^®^ Glaucoma Valve (New World Medical, Inc., Rancho Cucamonga, CA, USA) (AGV), Ex-PRESS^®^ Glaucoma Filtration Device (Alcon Laboratories, Inc., Fort Worth, TX, USA) (Minishunt), iStent^®^ (Glaukos Corporation; Aliso Viejo, CA, USA) (iStent), Hydrus^®^ Microstent (Ivantis, Inc., Irvine, CA, USA) (Hydrus), XEN^®^ 45 Gel Stent (Allergan, Inc., Dublin, Ireland) (Gel Stent), PRESERFLO^TM^ MircoShunt (Santen; Osaka, Japan) (MicroShunt). Trabeculectomy, Minishunt, and AGV were performed with peribulbar anesthesia. Topical anesthesia was used for the iStent and Gel Stent devices, while sub-Tenon’s capsule anesthesia was administered for the Hydrus and MircoShunt procedures. Trabeculectomy, AGV and Minishunt were considered traditional glaucoma surgery, iStent and Hydrus devices were categorized as MIGS, and Gel Stent and MicroShunt devices were classified as MIBS. According to the year in which surgery was performed, patients were divided into 2 groups: Group A, comprising patients who underwent glaucoma surgery between January and December 2011, and Group B with patients who received surgery between January and December 2021.

### 2.2. Statistical Analysis

Statistical analysis was performed using SPSS Statistics version 26 (IBM Corporation, Armonk, NY, USA). The Kolmogorov–Smirnov test was employed to assess the normal or skewed distribution of all variables. Data were presented as either the mean ± standard deviation (SD) or median and interquartile range, as appropriate. Quantitative variables between the two groups were compared using the independent *t*-test or the corresponding non-parametric Mann–Whitney U test, as applicable. The chi-squared test and Fisher’s exact test, as needed, was used for the comparison of categorical variables. A two-tailed *p*-value < 0.05 was considered statistically significant for all analyses.

## 3. Results

A total of 489 patients (489 eyes) were enrolled for the study, but 455 were finally included in the analysis due to the lack of ophthalmic information in the remaining 44 patients. Group A consisted of 230 eyes (117 males and 113 females) and Group B of 225 eyes (128 males and 97 females). The demographic and ophthalmic characteristics of patients are reported in Table 1. There were no statistically significant differences in terms of sex, central corneal thickness, number of phaco-combined surgeries, pseudophakia, or the use of oral acetazolamide between Groups (*p* > 0.05). However, in Group A, the patients were significantly older than those in Group B (*p* = 0.02) and had higher pre-surgical IOP values (*p* = 0.02). Notably, Group B showed a higher number of patients receiving surgery without using any hypotensive eye drops in the pre-operative period (*p* = 0.01).

The differences in glaucoma subtype classification, VF data, stage of disease, and surgical procedures performed between the two groups are presented in Table 2. There were no significant differences in the subtypes of glaucoma between the groups. Conversely, Groups A and B presented statistically significant differences for the VF mean defect (MD), which was higher in Group A (−16.4 ± 8.8) compared to Group B (−13.8 ± 8.7) (*p* < 0.01). On the other hand, the PSD value did not differ between the groups (*p* = 0.07). Of note, the number of patients with early VF damage, according to HPAc, was higher (two-fold) in Group B compared to Group A (*p* = 0.02). Contrary, Group A included a higher number of patients (almost 75%) with severe VF defects (*p* < 0.01). Group A included 19 trabeculectomies, 179 Minishunts, 13 AGVs, 5 iStents, and 14 Hydrus, while in Group B, there were 37 trabeculectomy, 50 Minishunt, 7 AGV, 68 Gel Stent, and 63 MicroShunt procedures. The overall number of MIGS and MIBS significantly differed between the two groups: 19 MIGSs in 2011 (5 iStents and 14 Hydrus) and 131 MIBSs in 2021 (68 Gel Stents and 63 MicroShunts), with none of the patients receiving ab interno MIGS. For patients at a severe stage of disease receiving traditional surgery, there were no statistically significant differences in MD values (Group A: −17.3 ± 8.7 decibel (dB); Group B: −17.2 ± 8.6 dB, *p* = 0.88) and PSD values (Group A: 9.2 ± 3.5; Group B: 8.7 ± 3.4, *p* = 0.25) between 2011 and 2021.

**Figure 1:**
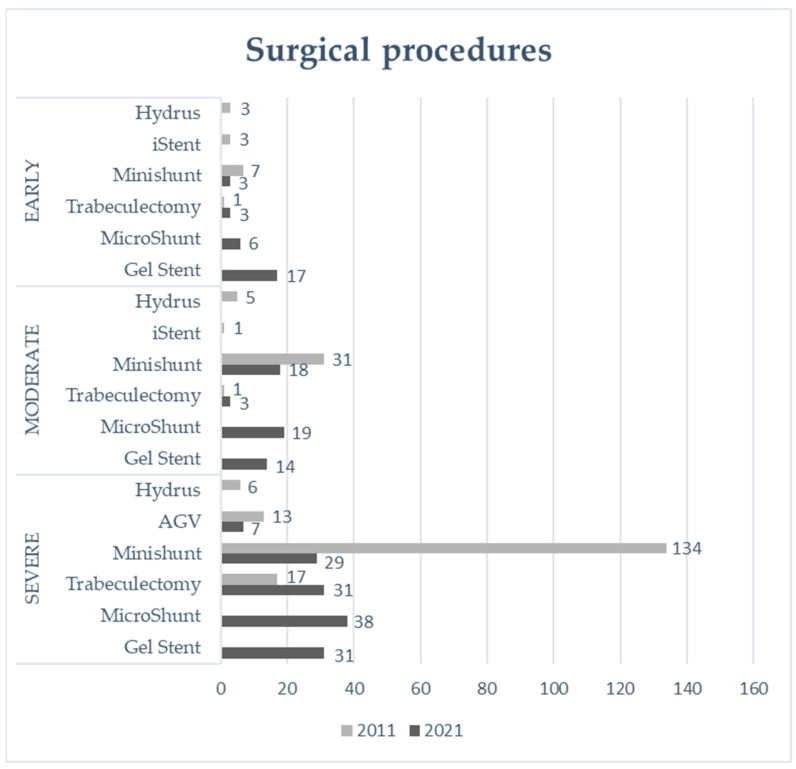
Distribution of surgical procedures between Group A (year 2011) and Group B (year 2021) according to the glaucoma stage. OHT: ocular hypertension; Trabeculectomy: trabeculectomy with mytomicin-C; Minishunt: Ex-PRESS^®^ glaucoma filtration device; AGV: Ahmed^®^ Glaucoma Valve; iStent: iStent^®^ Trabecular MicroBypass Stent; Hydrus: Hydrus^®^ Microstent; Gel Stent: XEN^®^ 45 Gel Stent; MicroShunt: PRESERFLO^TM^ MicroShunt.

Figure 1 reports the surgical procedures of the two groups, categorized according to the pre-operative VF.

**Figure 2:**
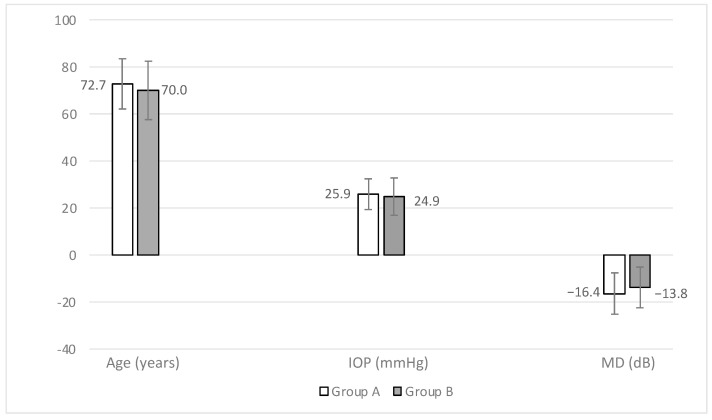
Age, IOP, and MD visual field parameters of the two Groups. Group A: 2011; Group B: 2021; IOP: intraocular pressure; MD: mean deviation.

Figure 2 summarizes the age (years), IOP (mmHg), and MD (dB) visual field parameter of the two Groups which represent the most significantly preoperative differences.

Table 3 summarizes the glaucoma surgical procedures in Groups A and B based on VF staging. In 2021, MIBS outpaced conventional surgical approaches for the early stage of glaucoma (*p* ≤ 0.01), whereas traditional surgeries were more frequently performed in severe cases (*p* = 0.02).

## 4. Discussion

The risk of glaucomatous damage progression should be evaluated by ophthalmologists considering multiple variables such as age, life expectancy, quality of life, central corneal thickness, IOP level, and baseline VF damage. Eyes with the worst VF damage at baseline and a clinical profile indicating a higher risk of progression should be more aggressively treated to reach a lower IOP target [7].

At our center, with the introduction of new less invasive surgical procedures, especially MIBS, the baseline ophthalmic characteristics of patients undergoing glaucoma surgery have significantly changed over the past decade. This ten-year period was selected for analysis because MIGS procedures became available at our center in 2011, and a decade provides a valid timeframe to evaluate the adoption and impact of various surgical techniques in an evolving technological landscape.

Overall, the patients treated in 2021 were younger (more than 2 years), with a more contained VF defect (15% lower MD) and lower IOP values than those treated in 2011. Conversely, ten years ago, the number of patients approaching surgery with severe VF defects and higher IOP values was significantly higher. Additional key emerging aspects are represented by the complete decline of the number of MIGS performed in 2011 (iStent and Hydrus) and the significant rise in MIBS (Gel Stent and MicroShunt), which were unavailable in 2011. In fact, in 2021 MIBS represented 58.2% of the overall first-time glaucoma surgical procedures at our center.

Our data support the fact that the introduction of MIBS led to the anticipation of surgical timing in terms of treating glaucomatous patients with a more contained functional impairment. When analyzing our data, the percentage of patients receiving surgery at an early stage of disease (MD > −6 dB, HPAc) in 2021 was double compared to that in 2011 (12.9% vs. 6.1%). This could be attributed to the fact that MIBS, which is associated with a relatively lower complication rate compared to traditional filtration surgery, encourages clinicians to recommend surgery at an earlier stage [13,14,15].

MIBS procedures played a significant role in changing baseline features of patients at our center because they were used for approximately 80% of cases presenting a contained VF defect. As such, MIBS increased the number of surgical procedures at an earlier stage of glaucoma. Moreover, the number of patients who did not receive any pre-operative topical hypotensive therapy was significantly higher in 2021 than that in 2011, with 11 cases and 2 cases from Groups B and A, respectively. This represents an interesting result considering that both the prolonged use of topical medications and complex therapy regimens lead to a well-defined comorbidity, the glaucoma therapy-related ocular surface disease, which is a strong risk factors for filtration surgery failure [18,19,20,21,22,23,24].

On the other hand, our study found that in recent years, the proportion of patients undergoing glaucoma surgery with a severe VF defect has decreased by nearly 15% (from 74.3% to 60.4%). This indicates that the introduction of MIBS is progressively shifting the surgical paradigm toward earlier intervention. With safer surgical procedures now available, traditional surgery is mainly reserved for more complex cases with higher functional impairment and for which achieving a very low target IOP is essential. However, due to the proven IOP-lowering efficacy and safety profile of MIBS, these procedures are also a viable option for severe cases, where safety is the primary concern [25,26].

A direct consequence of this aspect is that the overall number of traditional glaucoma surgeries decreased by more than 50% in the last years (from 211 to 94), remaining the preferred choice in more challenging cases also in 2021. Trabeculectomy remains the gold standard surgical treatment for uncontrolled glaucoma and is still widely adopted [6,8]. Nevertheless, it may lead to severe early post-operative complications such as hypotony, hyphema, and choroidal detachment, as well as long-term complications such as endophthalmitis and bleb-related problems [9,10]. The integration of these aspects with the availability of new effective and safer surgical approaches in the last years supports a general trend that is emerging over the past decades, whereby the field of indication of standard filtration surgery is going to be reconsidered. As found by Arora KS et al., the number of trabeculectomies performed in the United States decreased by more than 50% between 2003 and 2012, although it remained the most frequent penetrating glaucoma procedure adopted [27]. In a further study, Rathi et al. reported a decline in the number of trabeculectomies performed over the years, with 60–80% of this procedure being carried out only by glaucoma sub-specialists [28]. Moreover, the same study reported an impressive shift of traditional surgery toward the use of MIGS and MIBS, which increased by 426% from 2012 to 2016. Yang SA et al. reported a reduction in the number of traditional glaucoma surgeries, including trabeculectomy and glaucoma drainage device implantation, from 16,215 to 13,701 within the period between 2013 and 2018 in the United States [29]. These data are generally align with our results and confirm the progressive reshaping of the surgical paradigm in glaucoma [28]. However, in this study, we considered only patients who underwent their first surgery, rather than the total number of glaucoma surgeries performed annually.

As the side results of this study, we mentioned a significant decrease in the number of Minishunt implantations, which declined by almost 40%, from 179 in 2011 to 50 in 2021. This also confirms the trend discussed earlier, as the reduction in Minishunt implants in 2021 can be largely explained by the advent of MIBS. In addition, a debate on the literature is still ongoing about trabeculectomy and Minishunt post-operative results. Some authors suggested no significant differences between the two techniques in terms of IOP, complications and success rate, while others reported a higher rate of post-operative complications after trabeculectomy compared to Minishunt implantation [30,31,32,33].

When considering MIGS, our results clearly indicated a complete decline in its utilization. This could depend on several factors, including the nature of patients referring to our center, which are even more complex and often at an advanced stage of disease. But it could also depend on the availability of new minimally invasive procedures (MIBS) more effective than ab interno MIGS, which offers patients a better control of the IOP, especially at advanced disease stages [34].

The MIGS available at our center (Hydrus and iStent) are no longer performed due to the necessity to reach the target IOP with a surgical success and, at the same time, control the healthcare spending.

The Italian public healthcare system, the Servizio Sanitario Nazionale (SSN), provides universal support for all citizens across every region of the country. Each hospital operates with an annual budget to deliver healthcare services, and receives specific reimbursements from the Ministry of Health. However, the SSN has not yet been adjusted to cover the costs of these new devices, making it unlikely for hospitals to adopt MIGS. Initially, MIGS devices were used for patients with early-stage glaucoma, but due to their high cost, they were phased out in our hospital in favor of devices like Minishunt, XEN, and MicroShunt, which are more suitable for treating more advanced stages of glaucoma. These bleb-related devices appear to be more effective in managing glaucoma progression, despite requiring many postoperative controls due to potential early and late bleb complications.

In contrast, the United States follows a different approach to glaucoma management. In fact, according to Yang SA et al., the number of MIGS procedures significantly improved in the period between 2013 and 2018, from 7568 to 39,677. Notably the number of iStent device implantations increased from 13% to 40% and, in 2017, iStent accounted for 43.7% of all glaucoma surgical procedures performed in the United States [29]. The spread of many safer and less invasive techniques alternative to traditional glaucoma approaches such as GATT, Kahook Dual Blade, OMNI Surgical system, Trabectome, iStent, and Hydrus Miscrostent could lead to anticipating the surgical timing in glaucoma patients [12]. Unfortunately, all these devices are expensive, except for GATT which has a moderate cost as it does not require the introduction of a drainage device, making it a possible and a valid therapeutical option, especially in developing countries [35].

However, despite the introduction of MIGS and MIBS, ophthalmologists tend to reserve surgery as a last-resort strategy for more severe cases; moreover, the possibility to perform minimally invasive surgery almost exclusively at glaucoma referral centers represents an additional deterrent. Possibly due to the cost and limited availability of these devices, most ophthalmologists in Italy prefer a conservative approach with topical eye drop therapy over selective laser therapy or surgical treatment [36].

This study presents some limitations. Firstly, its retrospective nature introduces the possibility of selection bias and issues related to incomplete data. The baseline data of patients were collected solely for the purpose of comparing and demonstrating how our surgical strategy has evolved, and thus, a long-term post-operative evaluation of the efficacy and safety was not conducted. Second, we did not consider all currently available MIGS, since for economic or local approval reasons, we do not have access to all devices. Third, the extensive use of Minishunt at our centre in the past decades (due to the preferences of surgeons) could have hypothetically created a bias in what is really happening to filtration surgery. In fact, the global decline in the number of standard filtration surgery could in part depend on the progressive disuse of these devices.

## 5. Conclusions

In conclusion, based on our results, from 2011 to 2021, patients receiving their first glaucoma surgery are younger, with a lower IOP and less severe disease, with an increased use of MIBS procedures. It seems that the rise in MIBS is changing our approach to surgery and it will be interesting to determine through future studies whether this trend will continue and have a significant impact on the disease management. This study could be considered a preliminary one for further development of prospective and randomized clinical trials which have the aim to confirm the role and positioning of MIBS in the glaucoma treatment paradigm.

## Figures and Tables

**Table 1:** Demographic and ophthalmic characteristics of the Groups.

	Group A	Group B	*p*
Population	230	225	
Age ± SD (years)	72.7 ± 10.7	70.0 ± 12.4	**0.02**
Sex (*n*; %)			0.22
M	117 (50.9)	128 (56.9)	
F	113 (49.1)	97 (43.1)	
CCT ± SD (microns)	519.4 ± 37.4	526.5 ± 42.4	0.06
IOP ± SD (mmHg)	25.9 ±6.6	24.9 ± 7.8	**0.02**
Stand-alone (*n*; %)	141 (61.3)	139 (61,8)	0.99
Phaco-combined surgery (*n*; %)	89 (38.7)	86 (38.2)	0.99
Pseudophakia (%)	104 (45.2)	96 (42.7)	0.45
IOP-lowering active compound (*n*; %)			
0	2 (0.9)	11 (4.9)	**0.01**
1	11 (4.7)	15 (6.7)	0.37
2	54 (23.5)	48 (21.3)	0.30
3	97 (42.2)	99 (44.0)	0.69
4	66 (28.7)	52 (23.1)	0.17
Oral acetazolamide (*n*; %)	79 (34.3)	60 (26.7)	0.08

Group A: 2011; Group B: 2021; SD: standard deviation; M: male; F: female; CCT: central corneal thickness; IOP: intraocular pressure; *n*: number; in bold statistically significant values.

**Table 2:** Glaucoma subtype, visual field data, and surgical procedure distribution between the groups.

	Group A	Group B	*p*
Glaucoma subtypes (*n*; %)			
POAG	122 (53.0)	131 (58.3)	0.26
PACG	22 (9.6)	12 (5.3)	0.11
PXG	37 (16.1)	30 (13.3)	0.43
PG	16 (7.0)	15 (6.7)	0.99
NTG	13 (5.7)	14 (6.2)	0.85
NVG	9 (3.9)	6 (2.7)	0.60
OHT	7 (3.0)	14 (6.2)	0.12
Uveitic glaucoma	4 (1.7)	3 (1.3)	0.99
Visual field data			
MD ± SD	−16.4 ± 8.8	−13.8 ± 8.7	**<0.01**
PSD ± SD	8.9 ± 3.6	8.2 ± 3.9	0.07
HPA classification (*n*; %)			
Normal	7 (3)	6 (2.7)	0.98
Early	14 (6.1)	29 (12.9)	**0.02**
Moderate	38 (16.5)	53 (23.6)	0.06
Severe	171 (74.3)	137 (60.8)	**<0.01**
Surgical procedures (*n*; %)			
Trabeculectomy	19 (8.3)	37 (16.5)	**<0.01**
Minishunt	179 (77.8)	50 (22.2)	**<0.01**
AGV	13 (5.6)	7 (3.1)	0.19
iStent	5 (2.2)	0 (0.0)	**<0.01**
Hydrus	14 (6.1)	0 (0.0)	**<0.01**
Gel Stent	0 (0.0)	68 (30.2)	**<0.01**
MicroShunt	0 (0.0)	63 (28.0)	**<0.01**

Group A: 2011; Group B: 2021; POAG: primary open-angle glaucoma; PACG: primary angle-closure glaucoma; PXG: pseudoexfoliation glaucoma; PG: pigmentary glaucoma; NTG: normal-tension glaucoma; NVG: neovascular glaucoma; OHT: ocular hypertension; SD: standard deviation; MD: mean deviation; PSD: pattern standard deviation; HPA: Hodapp–Parrish–Anderson; Trabeculectomy: trabeculectomy with mytomicin-C; Minishunt: Ex-PRESS^®^ glaucoma filtration device; AGV: Ahmed^®^ Glaucoma Valve; iStent: iStent^®^; Hydrus: Hydrus^®^ Microstent; Gel Stent: XEN^®^ 45 Gel Stent; MicroShunt: PRESERFLO^TM^ MicroShunt; in bold statistically significant values.

**Table 3:** Glaucoma surgical procedures in Groups A and B.

**Group A**	**Surgical Procedure**	**MIGS**	**Traditional Surgery**	***p*-Value**
OHT	7 (3.1)	1 (5.2)	6 (2.8)	0.56
Early	14 (6.1)	6 (31.6)	8 (3.8)	**<0.01**
Moderate	38 (16.5)	6 (31.6)	32 (15.2)	0.07
Severe	171 (74.3)	6 (31.6)	165 (78.2)	**<0.01**
Total	230 (100)	19 (100)	211 (100)	
**Group B**	**Surgical Procedure**	**MIBS**	**Traditional Surgery**	***p-*** **Value**
OHT	6 (2.7)	6 (4.6)	0 (0.0)	**<0.01**
Early	29 (12.9)	23 (17.5)	6 (6.4)	**<0.01**
Moderate	54 (24.0)	33 (25.2)	21 (22.3)	0.62
Severe	136 (60.4)	69 (52.7)	67 (71.3)	**0.02**
Total	225 (100)	131 (100)	94 (100)	

Group A: 2011; Group B: 2021; OHT: ocular hypertension; MIGS: minimally invasive glaucoma surgery; MIBS: minimally invasive bleb surgery. (Numbers in parentheses stand for percentages). In bold statistically significant values.

## Data Availability

The data presented in this study are available on request from the corresponding author due to privacy.
